# Enhanced optical and electrochemical properties of FeBTC MOF modified TiO_2_ photoanode for DSSCs application

**DOI:** 10.1038/s41598-024-61701-3

**Published:** 2024-05-17

**Authors:** William Moloto, Pontsho Mbule, Edward Nxumalo, Bulelwa Ntsendwana

**Affiliations:** 1 Institute for Nanotechnology and Water Sustainability, CSET, University of South Africa, Johannesburg, 1710 South Africa; 2 Department of Physics, CSET, University of South Africa, Johannesburg, 1710 South Africa; 3 Energy, Water, Environmental and Food Sustainable Technologies (EWEF-susTech), Johannesburg, 1709 South Africa

**Keywords:** TiO_2_, TiO_2_–FeBTC nanocomposites, Photoefficiency, DSSCs, Electrochemical impedance, Sensors, Photonic devices, Electrochemistry

## Abstract

In this work, iron based 1, 3, 5-tricarboxylic acid (FeBTC) was prepared via microwave-assisted method and incorporated into TiO_2_ via ultrasonic assisted method. The TiO_2_–FeBTC nanocomposites were characterized by XRD, FTIR, Raman, BET, FESEM, HRTEM, TGA, UV‒vis DRS and PL to understand their crystallographic, surface morphology, and optical characteristics. The Raman spectra showed a blue shift of E_g_, A_1g_, and B_1g_ peaks upon incorporation of FeBTC MOF onto TiO_2_. HRTEM and XRD analysis confirmed a mixture of TiO_2_ nanospheres and hexagonal FeBTC MOF morphologies with high crystallinity. The incorporation of FeBTC onto TiO_2_ improved the surface area as confirmed by BET results, which resulted in improved absorption in the visible region as a results of reduced bandgap energy from 3.2 to 2.84 eV. The PL results showed a reduced intensity for TiO_2_–FeBTC (6%) sample, indicating improved separation of electron hole pairs and reduced recombination rate. After fabrication of the TiO_2_–FeBTC MOF photoanode, the charge transfer kinetics were enhanced at TiO_2_–FeBTC MOF (6%) with Rp value of 966 Ω, as given by EIS studies. This led to high performance due to low charge resistance. Hence, high power conversion efficiency (PCE) value of 0.538% for TiO_2_–FeBTC (6%) was achieved, in comparison with other loadings. This was attributed to a relatively high surface area which allowed more charge shuttling and thus better electrical response. Conversely, upon increasing the FeBTC MOF loading to 8%, significant reduction in efficiency (0.478%) was obtained, which was attributed to sluggish charge transfer and fast electron–hole pair recombination rate. The TiO_2_–FeBTC (6%) may be a good candidate for use in DSSCs as a photoanode materials for improved efficiency.

## Introduction

Photovoltaics (PVs) are one of the important devices that enable the conversion of sunlight into energy. Photovoltaics can be categorised into three generations. The first generation is made of the crystalline and polycrystalline silicon solar cells, and are fabricated using very expensive materials and thus, difficult to acquire. The second generation utilizes copper indium gallium selenide (CIGS), cadmium telluride (CDTe), amorphous silicon (a-Si:H) and gallium arsenide (GaAs)^1,2^. They are much cheaper compared to the first generation PVs, however, there has been toxicity concerns due to the use of transition elements^2^. The third generation solar cells have emerged as promising alternatives due to their low cost of fabrication^1,3,4^. In fact, research has suggested that they have the potential to replace conventional fossil fuels based on the materials use and affordable processes of fabrication^5^. Photovoltaics includes quantum dots, organic, perovskite and dye sensitized solar cells (DSSCs)^2,6–8^. The third-generation solar cells are at a developmental stage and have shown promising potential. However, the efficiency of DSSCs is still low compared to other solar cells and more research has been devoted in improving their performance through utilisation of a wide range of cheaper materials and processes of fabrications^3,5,9^. Noorasid et al. surveyed suitable methods of deposition for the preparation of flexible DSSCs^9^. This will allow the technology to be more affordable and accessible in many households. One of the most important components of DSSCs is the photoanode, since it is responsible for generation of the electrons upon light illumination^3^. Therefore, it is important to prioritise the development of photoanodes with optimal efficiencies for DSSCs applications. The most commonly used photoanode material is TiO_2_, due to its attractive properties such as abundance, increased stability, non-toxicity, and the presence of mesopores in their structure^10–12^. However, TiO_2_ has several drawbacks, including large band gap energy, which limits its absorption capacity to UV region. This and low charge separation and charge transportation which further leads to fast recombination rates that eventually lead to a reduced photo-conversion efficiency.

The improvement of TiO_2_ photoanode using other materials such as Al_2_O_3_ and carbon nanomaterials (graphene oxide, carbon nanotubes and carbon dots) is well documented in literature^13–15^. For example, Choi et al. used Al_2_O_3_ to modify the surface of TiO_2_ using solgel method^16^. It was found that uniform distribution on surface of TiO_2_ increased the short circuit photocurrent and hence the overall energy conversion efficiency. The increase in light scattering upon addition of Al_2_O_3_ induced improved performance of DSSCs. Other researchers used reduced graphene oxide (rGO) to modify TiO_2_ to improve the performance of DSSCs^2^. The incorporation of rGO onto TiO_2_ showed higher dye adsorption resulting in lower internal resistance and effect faster electron kinetics. The power conversion efficiency (PCE) value was improved to 6.69%, indicating almost 11% increase compared to the PCE of pure TiO_2_ 5.97%^13^. Carbon quantum dots (CQDs) with their quantum confinement have also been used to modify TiO_2_ photoanodes^4^. The incorporation of CQDs improved the kinetics and light harvesting by supressing bulk recombination rate of photogenerated electron–hole pairs. The photocurrent increased by 11.72 times compared to pure TiO_2_ photoanode^15^.

On the other hand, metal organic frameworks (MOFs) have emerged as suitable materials that can be used as photoanode materials and sensitizers in DSSCs application due to their improved charge transportation and enhance light absorption (via organic linker engineering)^17^. Metal organic frameworks are coordinated compounds made up of organic ligands and polynuclear metal nodes that enable opening of a porous crystalline framework structure. Thus, they exhibit high specific surface area, up to 7000 m^2^/g^−1^ and they have been extensively used in various applications such as gas storage^18^, biomedical^19^, catalysis^20^, gas–vapor separation^21^, energy and electronics^22^. In DSSCs applications, the high surface area materials such as MOFs are important as they allow more dyes to be adsorbed on the surface of the semiconductor material and therefore improve electron transportation, thereby hampering electron–hole recombination rates. Furthermore, MOFs exhibit suitable bandgap energy in the visible region, which satisfy the principle of photovoltaic device. It has been demonstrated that through metal–ligand charge transfer interaction, MOFs properties can be further enhanced via rational composition engineering^17,23,24^. Hence, they have shown capabilities when either used directly as photoanode materials^25^, or photosensitizers and electron shuttles to improve photoactivity and electron transportation^26^. However, the catalytic activity of MOFs depends on the metal type used. Metal ions with *d* shell such as Nickel (Ni), Cu (Copper) and Cobalt (Co) are effective in tuning the bandgap energy^27,28^. The iron-based MOFs have also showed better absorbing light properties in the visible region (380–800 nm). This aspect suggests that the iron-based MOFs can be used as photosensitizers^29^. Additionally, they also exhibit good catalytic activity which can aid the charge transfer kinetics^30,31^. Hence, in this study, we report the use of FeBTC MOF to modify the properties of TiO_2_ for its potential use in DSSCs applications. The use of BTC (benzene-1, 3, 5-tricarboxylic acid) ligand is due its ease of dissolution which also requires no harmful solvent. Additionally, the ligands show good thermal stability, and this may allow the materials to be stable at higher temperatures. The incorporation of Fe-based MOFs in TiO_2_ photoanodes is expected to improve the charge separation of electron hole pairs by retarding recombination rate.

## Experimental procedure

### Materials

Titanium isopropoxide (Ti([OCH(CH_3_)_2_]_4_, 97%), trimesic acid (benzene-1,3,5-tricarboxylic acid) (H_3_BTC, 95%), ethanol (96%), Isopropyl alcohol (C_3_H_8_O, 99.9%) polyethylene glycol (PEG 400) C_2_nH_4_n + _2_On + 1, < 99%), ammonium hydroxide (NH_4_OH, 25%), NaOH, 98%, iron chloride (FeCl_3_·6H_2_O, 97%) were all purchased from Sigma-Aldrich (South Africa) and used without any further purification.

### Synthesis of titanium dioxide (TiO_2_)

The synthesis procedure for TiO_2_ is described in our previous report^12^. Typically, 15 mL of titanium isopropoxide and 45 mL of isopropyl alcohol were transferred into a 1000 mL beaker, where 250 mL of distilled water was used as hydrolysis catalyst. After adjusting the pH to 11.5 using NH_4_OH, the mixture was stirred at 500 rpm at room temperature (27 °C) to create a homogenous solution. To catalyse the reaction, the solution mixture was heated for 20 h at 60 °C. The peptization process was followed by reduction in solution to about 30 mL to allow the white viscous suspension to be produced. The obtained white gel solution was washed 3 times using ethanol and then twice with distilled water. After air drying for 24 h, the material was further dried in the oven at 100 °C for 12 h. The TiO_2_ powder was obtained by annealing for 2 h at 450 °C in a muffle furnace.

### Synthesis of FeBTC MOF

The FeBTC MOF was synthesized via microwave assisted method. In a beaker containing 20 mL of polyethylene glycol (PEG-400), 40 mg of FeCl_3_·6H_2_O was added and allowed to stir for 15 min to create a suspension. In a 20 mL separate beaker, 4.2 mg of trimesic acid was added to 0.01 M sodium hydroxide (NaOH) solution. The two solutions were then mixed and transferred in a glass vial. The glass vial was then sealed with a Teflon lid and put in the microwave reaction vessel. The mixture was allowed to react for 30 min using maximum power of 500 W. After the reaction was complete, the vessel was allowed to cool to room temperature before the resultant suspension was washed with distilled water (3 times) and then with ethanol (2 times) using a centrifuge instrument. The powder was then dried in air for 48 h and thereafter dried for 12 h in an oven at 70 °C.

### Synthesis of TiO_2_–FeBTC nanocomposites

The synthesis of the nanocomposite materials was carried out using ultrasonic assisted method. Firstly, TiO_2_ (1.5 g) powder was sonicated for 10 min in a beaker containing 10 mL of ethanol. In a separate beaker containing 10 mL of distilled water, FeBTC MOF (2 mg) was sonicated for 10 min. The two mixture were then transferred into a microwave reaction vessel for 30 min and allowed to cool to room temperature. The resulting suspension was washed in ethanol (3 times) and also in distilled water (2 times) using a centrifuge instrument. The powder was allowed to dry for 48 h in air and then dried in the oven for further 12 h at 70 °C. The powder was then sintered at 300 °C for 45 min. The TiO_2_–FeBTC (2, 4, 6, and 8%) nanocomposites were obtained by varying the loadings of FeBTC MOF.

### Characterization techniques

Various instrumental techniques were used to examine the properties of the as-synthesized materials. FTIR spectra PerkinElmer FTIR spectrometer Frontier (spectrum 100 spectrometer) using KBr pellet method at 4 cm^−1^ resolution was used to study the presence of functional groups. A Witec Raman spectrometer (Alpha 300, TS 150) at 100× magnification and laser power source of 532 nm was used to collect Raman spectra. A Rigaku SmartLab X-ray diffractometer with Cu-Kα (λ = 0.154059 nm) radiation at 2°/min was used to perform XRD analysis. X-ray photoelectron spectroscopy (XPS) analysis was carried out using Thermo ESCalab 250 Xi spectrometer, with Al Kα (1486.7 eV) monochromatic X-ray radiation source which was calibrated by carbon deposit C (1 s) binding energy at 284.8 eV.

A JEOL JSM-7800F field emission scanning electron microscopy (FESEM) coupled with Oxford Aztec 350 X-Max80 electron dispersive spectroscopy (EDS), was used to record surface morphology and elemental analysis. High resolution TEM Tecnai G2F2O X-Twin MAT (Eindhoven, Netherlands) operating at 200 kV was used to analyse the internal morphology of the materials. Brunauer–Emmett–Teller (BET) nitrogen adsorption–desorption (Micromeritics Tristar Surface area and Porosity Analyser) was used to obtain the surface area, pore size and volume. To study the thermal stability of the materials under the inert environment from 35 to 900 °C at 10.00 °C/min heating rate a Perkin Elmer Pyris 1 Thermogravimetric analyser (TGA) was used. The optical properties were evaluated using PerkinElmer UV/Vis/NIR spectrometer Lambda 1050 from 200 to 800 nm wavelength range. A Horiba Fluorolog-3 Jobnyvon at the excitation wavelength of 325 nm was used to record photoluminescence spectra.

### Fabrication of the photoanodes

**Figure 1:**
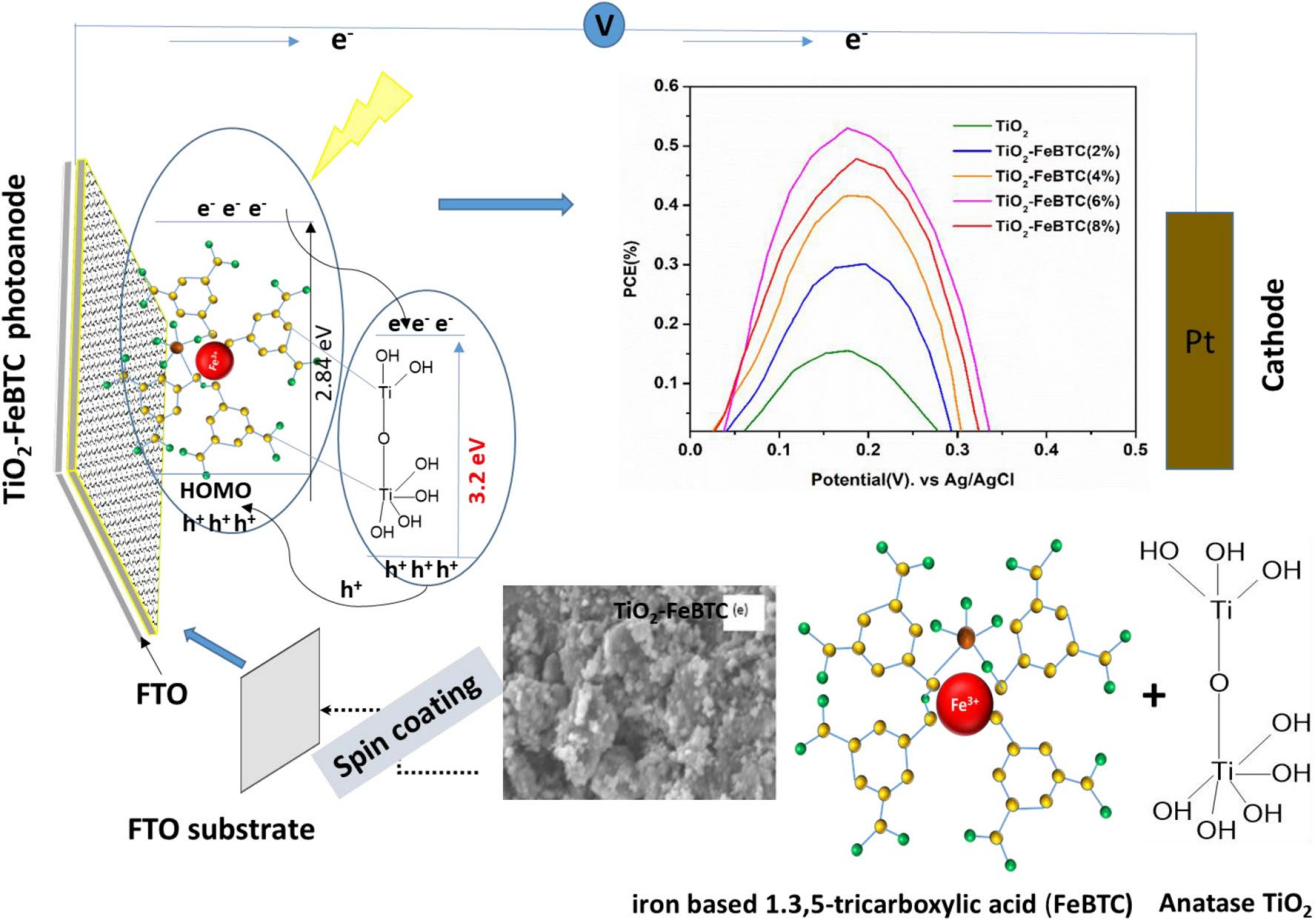
Schematic of the fabrication of TiO_2_–FeBTC photoelectrode.

Firstly, the FTO glass substrates (13.7 Ω) were soaked in a beaker containing soap and ultrasonicated for 15 min and rinsed with distilled water. They were further ultrasonicated in absolute ethanol for 15 min and rinsed with distilled water. The substrate was then baked in the furnace at 500 °C to remove any organic residues and thereafter stored in a clean desiccator to prevent any further contamination. The paste was obtained by dissolving 2 g of powder in 1.5 mL of dimethyl sulfoxide (DMSO) and allowed to sonicate for 10 min. The resultant paste was dropped on a clean FTO substrate and spin coated at 6000 rpm for 80 s. The coated films were then heated on the stove for 30 min at 90 °C, after which the films were baked in the furnace at 450 °C for 30 min (Fig. 1).

### Photoelectrochemical studies

A computer-controlled potentiostat/galvanostat (galvanostat (Autolab, PGSTAT 302 N model, Metrohm, Swiss instruments) was used to obtained electrochemical data for the photoelectrodes. A three-set-up electrode, platinum foil, Ag/AgCl and prepared photoanodes (1.4 × 1.4 cm) were used as counter, reference and working electrodes respectively. An electrolyte containing 5 mM acetonitrile solution, 0.055 M LiI, 9 mM I_2_ and 0.85 M LiClO_4_ was used to carry the measurements. All EIS measurements were carried out at the frequency range of 1–100 kHz with voltage amplitude of 25 mV under the dark. To obtain photodensity measurements, a solar simulator equipped with a Xenon lamp and AM 1.5 filter under one-sun illumination (100 mW/cm^2^) was used.

## Results and discussion

### XRD analysis

**Figure 2:**
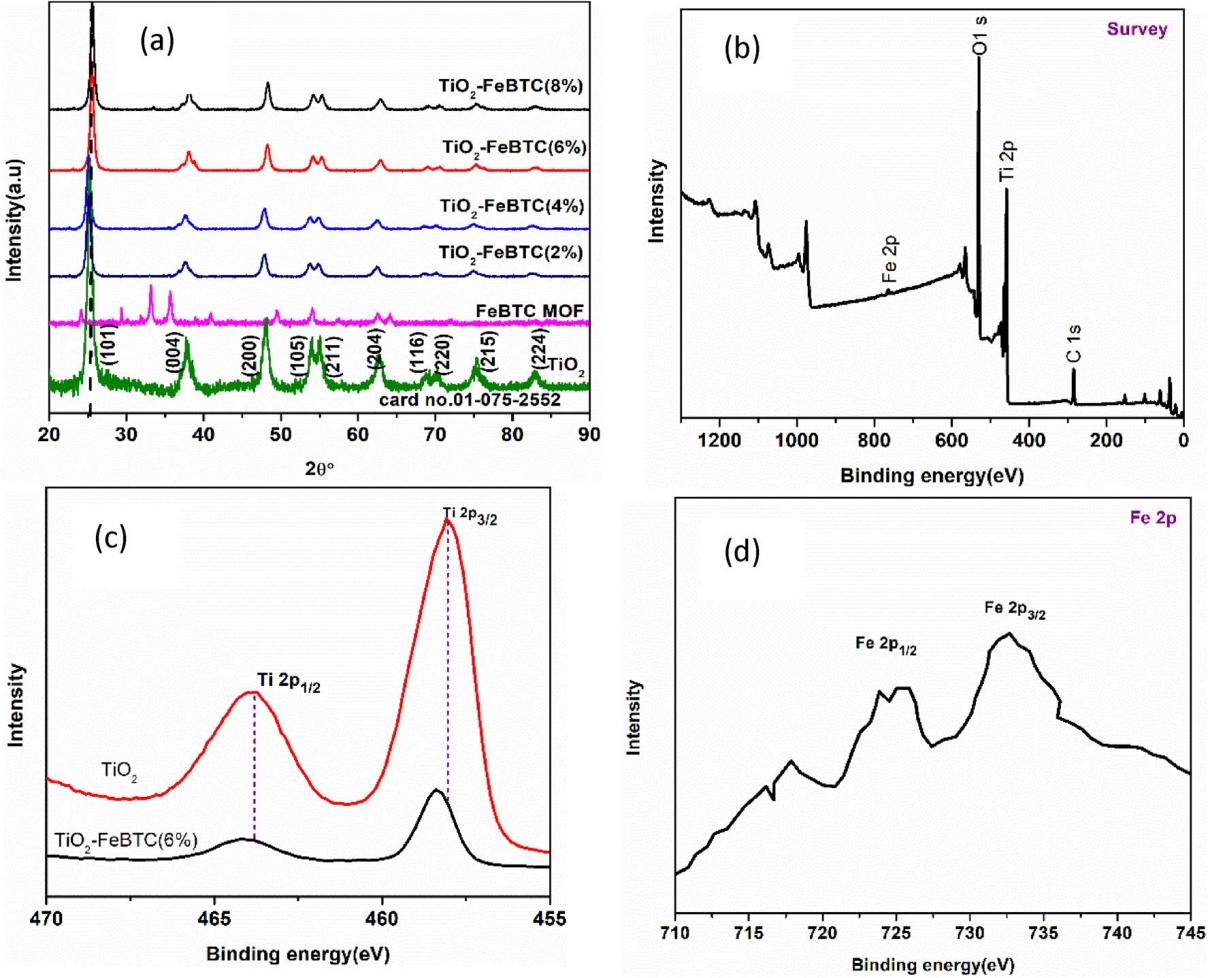
(**a**) XRD spectra of the as-synthesised materials (TiO_2_, FeBTC MOF and TiO_2_–FeBTC (2,4,6 and 8%) and XPS spectra: (**b**)) Wide spectra, (**c**) Ti 2p, and (**d**) Fe 2p.

To evaluate the crystallographic information, XRD analysis of as-synthesized samples was performed as presented in Fig. 2. The results showed that, TiO_2_ exhibits indexed diffraction peaks of a tetragonal anatase phase (peaks at 2Ɵ = 25.58° (101), 37.8.17° (004), 48.4° (200), 55.33° (105), 61.20° (204), 68.5° (220) and 76.1° (301) corresponding to the standard data, card no. 01-075-2552. These peaks were also observed in TiO_2_–FeBTC (2, 4, 6 and 8%) nanocomposite materials. However, there was a red shift with reduced peak intensity in all of the nanocomposite materials, which highlights the interaction between TiO_2_ and FeBTC. As it is reported elsewhere, the shifting of the peaks is related to structural relaxation upon the introduction of Fe-MOF on to TiO_2_ and this is due to changes in lattice parameters^12,32^.

The crystallite size, micro-strain and the D spacing results are given in Table 1. The crystallite size and micro-strain were estimated using the Eqs. (1) and (2) respectively:

$$D =\frac{0.9\lambda }{\beta cos\theta }$$

$$\varepsilon =\frac{\beta }{4tan\theta }$$

where θ is the glancing Braggs angle with the sample holder, λ = 0.15418 is the wavelength of CuKα radiation, and β is the line broadening at half the maximum intensity (FWHM). Accordingly, crystallite size ranged between 9.7–16 nm for the as-synthesized materials. There was a decrease in crystallite size and micro strain with FeBTC loading suggesting a change in crystallinity of TiO_2_ upon the addition of FeBTC MOF.

**Table 1:** Crystallite size, micro-strain and D-spacing of the as-synthesized materials.

Sample	Crystalline size	Micro-strain	D-spacing
TiO_2_	16.40	0.211	0.332
FeBTC MOF	9.70	0.091	0.387
TiO_2_–FeBTC (2%)	15.10	0.204	0.344
TiO_2_–FeBTC (4%)	14.65	0.196	0.346
TiO_2_–FeBTC (6%)	12.81	0.197	0.359
TiO_2_–FeBTC (8%)	11.94	0.194	0.343

To validate the chemical composition and oxidation state of the elements, XPS analysis was carried out as shown in Fig. 2b–d. The wide spectra in Fig. 2b showed the elemental constituents of pure TiO_2_ and TiO_2_–FeBTC (6%) material. In Fig. 2c the high resolution spectra of TiO_2_ showed the presence of two peaks at 463.8 and 458.1 eV, representing the Ti 2p_1/2_ and Ti 2p_3/2_ of the core level of Ti^4+^, respectively. In TiO_2_–FeBTC (6%), these peaks are located at 464.1 and 458.3 eV for Ti 2p_1/2_ and Ti 2p_3/2_. Additionally, the Fe 2p spectra in Fig. 2d showed two main peaks at 724 and 732 eV which are assigned to Fe 2p_3/2_ and Fe 2p_1/2_ in Fe-BTC MOF, respectively. These two peaks with the presence of satellite peak at 729 eV suggest that the Fe-BTC MOF exist in the trivalent (Fe(III)) state. The O1s spectra in TiO_2_–FeBTC (6%), demonstrated a slight shift in binding energy, indicating a formation of C–Ti–O bonding (Fig. S1). The shift in binding energy suggest a strong interaction between the FeBTC and TiO_2_, which might facilitate the electron transfer between the material upon light illumination and applied potential/current density.

### Morphological properties

#### SEM analyses of nanocomposite materials

**Figure 3:**
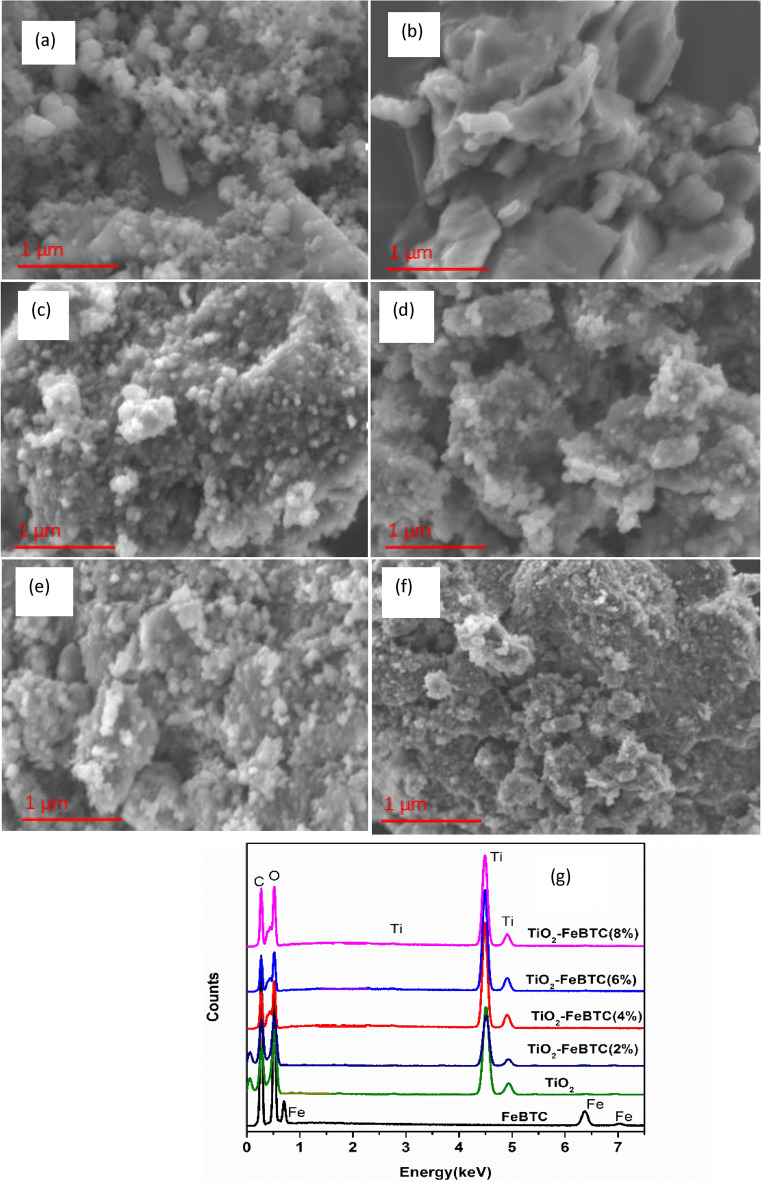
HRSEM images of (**a**) TiO_2_ nanoparticles, (**b**) Fe-BTC MOF, (**c**–**f**) TiO_2_–Fe-BTC (2, 4, 6% and 8%) and (**g**) corresponding EDS spectra.

SEM images of the as-synthesized materials are shown in Fig. 3. The SEM image of TiO_2_ in Fig. 3a shows small aggregated spheres of nanoparticles. The aggregation may be due to stirring during the gelation in the synthesis step. The FeBTC MOF in Fig. 3b is characterized by irregular particles of different shape and size. The SEM images of TiO_2_–FeBTC MOF nanocomposites (Fig. 3c–f) are characterized by TiO_2_ nanoparticles dispersed on the surface of larger FeBTC MOF nanoparticles. An increase in aggregation of nanoparticles, with an increase in FeBTC MOF loading was observed. The increase in aggregation may reduce the surface area of the materials and therefore hinder electron-charge transfer as there will be limited surface area for electron transfer reactions. Furthermore, EDS spectra in Fig. 3g showed the expected elements of TiO_2_ (Ti, O, and C) and Fe-BTC (Fe, C, and O) in the nanocomposites. The EDS of TiO_2_–FeBTC (2, 4, 6, and 8%) are characterized by dominant Ti, O and C elements. Fe element was not observed in the nanocomposites materials and this could be due to low concentration used during the synthesis step.

#### HRTEM analysis

**Figure 4:**
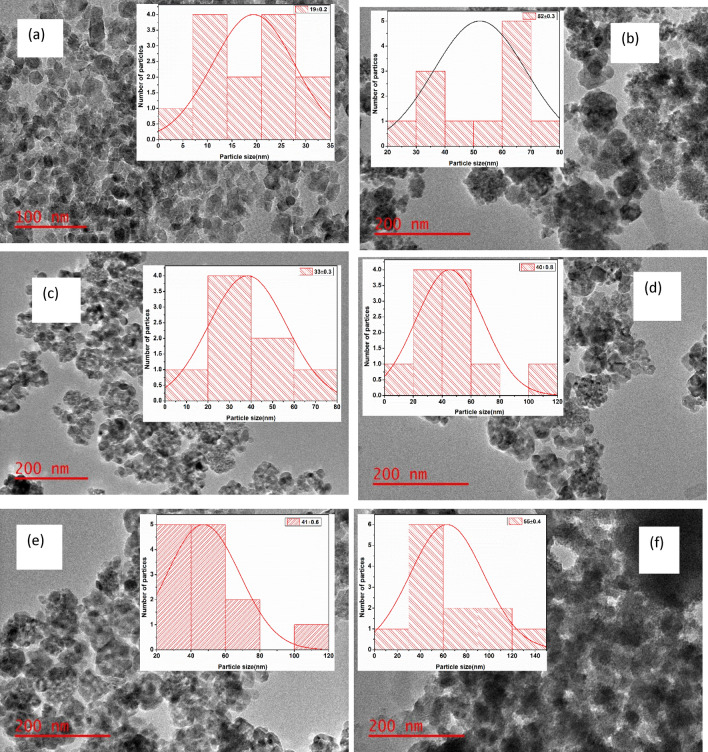
HRTEM images and particle size histograms of (**a**) TiO_2_, (**b**) FeBTC MOF, and (**c**) TiO_2_–FeBTC (2%), (**d**) TiO_2_–FeBTC (4%), (**e**) TiO_2_–FeBTC (6%) and (**f**) TiO_2_–FeBTC (8%).

The morphology of the as-synthesized materials was further evaluated by HRTEM. Image J software was used to estimate the particle size of the materials. Figure 4a shows HRTEM image of TiO_2_ which is characterized by aggregated nano-spheres with varying particle sizes, ranging between 4–35 nm. The variation of size was induced by the stirring during the gelation process^12^. The FeBTC MOF exhibited hexagonal shapes with poorly formed aggregated nanoparticles of different sizes as seen in Fig. 4b. The nanoparticles have a characteristic of moderate sorting which implies that the particle size variation is small. The particle size of FeBTC MOF ranged between 20–80 nm. The HTREM images for TiO_2_–FeBTC(2,4,6 and 8%) nanocomposites are shown in Fig. 4c–f respectively. The HRTEM images of the nanocomposites are characterized by a mixture of smaller TiO_2_ nanoparticles and much larger FeBTC MOF particles, attached to surface of each other. The FeBTC MOF nanoparticles are well dispersed on the surface of TiO_2_ and this can be seen in all nanocomposites. The increase in loading of FeBTC MOF is also evident as the number of larger particles are observed at higher loadings from Fig. Fig. 4c–f. Hence, the aggregation of particles is more prevalent in TiO_2_–FeBTC (8%) nanocomposite (Fig. 4f) and thus, leading to a reduced pore sizes. The reduction of pore volume and sizes is an undesired consequence as it can reduce the charge transportation of electrons and also impact electron collection by the photoanode half of DSSCs, which will result in higher recombination rate of electron–hole pairs.

#### N_2_ adsorption–desorption isotherms

The BET results in Table 2 show that TiO_2_ exhibited a low specific surface area of 106.75 m^2^/g, which is consistent with our previous findings^12^. However, upon incorporation of FeBTC MOF, the surface area of 636.53 m^2^/g, which was four times higher than that of TiO_2_ was obtained. This means that, incorporation of MOF on TiO_2_ matrix, considerably improves the specific surface areas. Consequently, he surface area was further improved with an increase of MOF loading by 2, 4, 6 and 8% reaching 188.5 m^2^/g, 256.71 m^2^/g, 328.54 m^2^/g, and 336.21 m^2^/g respectively (Table 2). Similarly, the pore size and pore volume of the TiO_2_–FeBTC nanocomposites also increased with an increase in MOF loading from 2 to 6%. However, the TiO_2_–FeBTC (8%) nanocomposite showed a decrease in pore volume and pore size, which is attributed to higher aggregation of the Fe-BTC MOF and TiO_2_ nanoparticles blocking the pores as evident from the SEM and HRTEM results.

**Table 2:** Band gap energy, BET surface area, pore size, pore volume and particle size of as-synthesized materials.

Sample	Band gap (eV)	BET surface area (m^2^/g)	Pore size (nm)	Pore volume (cm^3^)	Particle size (nm)
TiO_2_	3.21	106.75	3.5	0.36	19
FeBTC MOF	1.92	636.53	9.21	0.64	52
TiO_2_–FeBTC (2%)	2.91	188.50	3.7	0.35	33
TiO_2_–FeBTC (4%)	2.88	256.71	4.45	0.38	40
TiO_2_–FeBTC (6%)	2.85	328.54	7.2	0.44	41
TiO_2_–FeBTC (8%)	2.84	336.21	6.4	0.39	55

**Figure 5:**
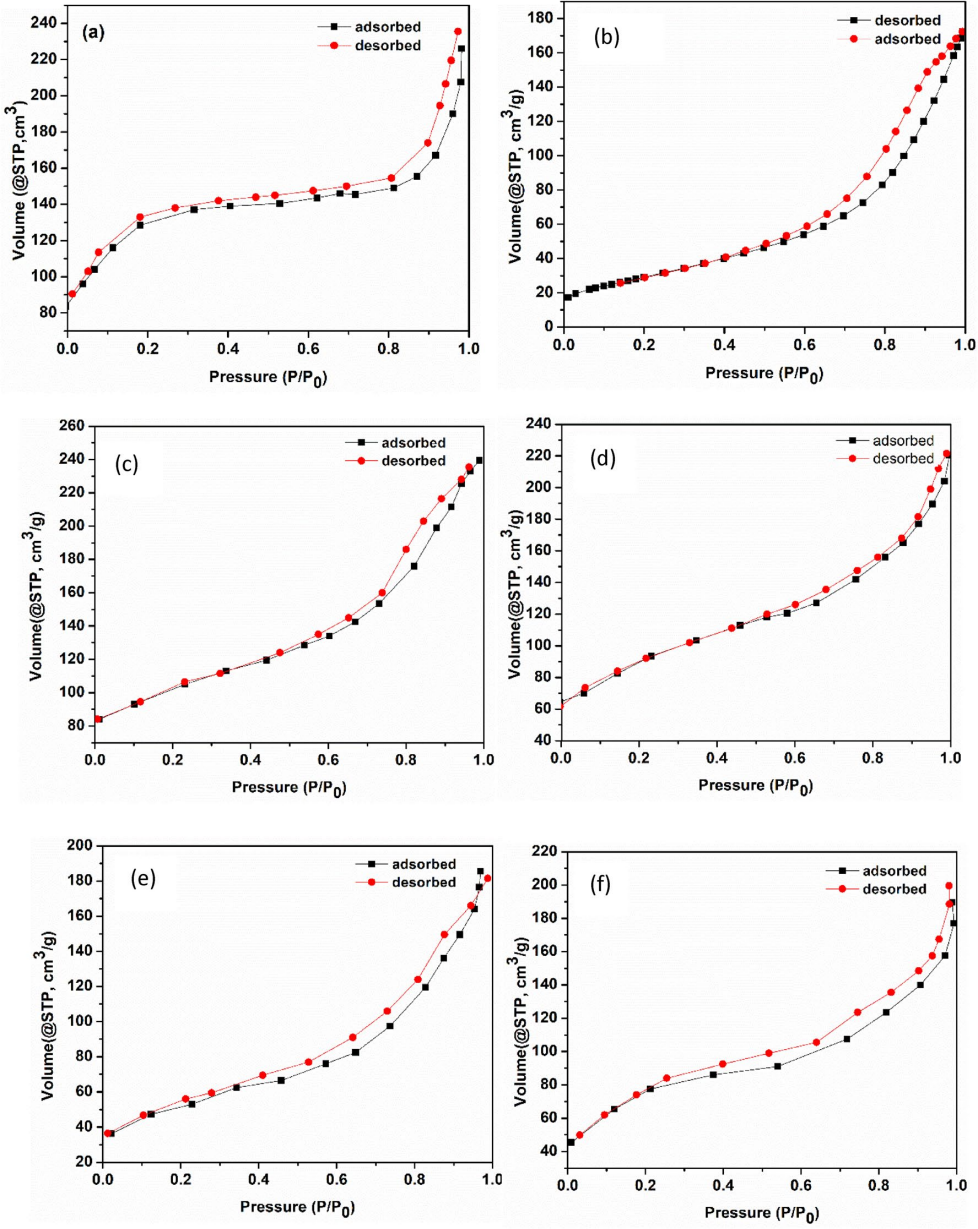
N_2_ adsorption–desorption isotherms of (**a**) TiO_2_, (**b**) FeBTC MOF, and (**c**) TiO_2_–FeBTC (2%), (**d**) TiO_2_–FeBTC (4%), (**e**) TiO_2_–FeBTC (6%) and (**f**) TiO_2_–FeBTC (8%).

Generally, DSSCs rely on the photoanode to absorb light and also generate electrons and therefore, the high surface area is an important property as it will aid in adsorption of the dye molecule on the surface of TiO_2_–FeBTC MOF and thereby improving sunlight absorption. The N_2_ adsorption–desorption isotherms corresponding to these samples are presented in Fig. 5a–f. The pure TiO_2_ in Fig. 5b and the nanocomposite materials in Fig. 5c–f, showed a type IV isotherm which suggest that there are stronger lateral interactions between adsorbed molecules. The hysteresis loop showed by the nitrogen adsorption–desorption isotherm of TiO_2_ confirms that the materials are mesoporous and resemble that of type H3 IUPAC (International Union of Pure and Applied Chemistry) classification. The FeBTC MOF isotherm in Fig. 5a, resembles that of type H1 which correlates with the porous material^33^.

#### Functional and thermal properties

##### Raman analysis

**Figure 6:**
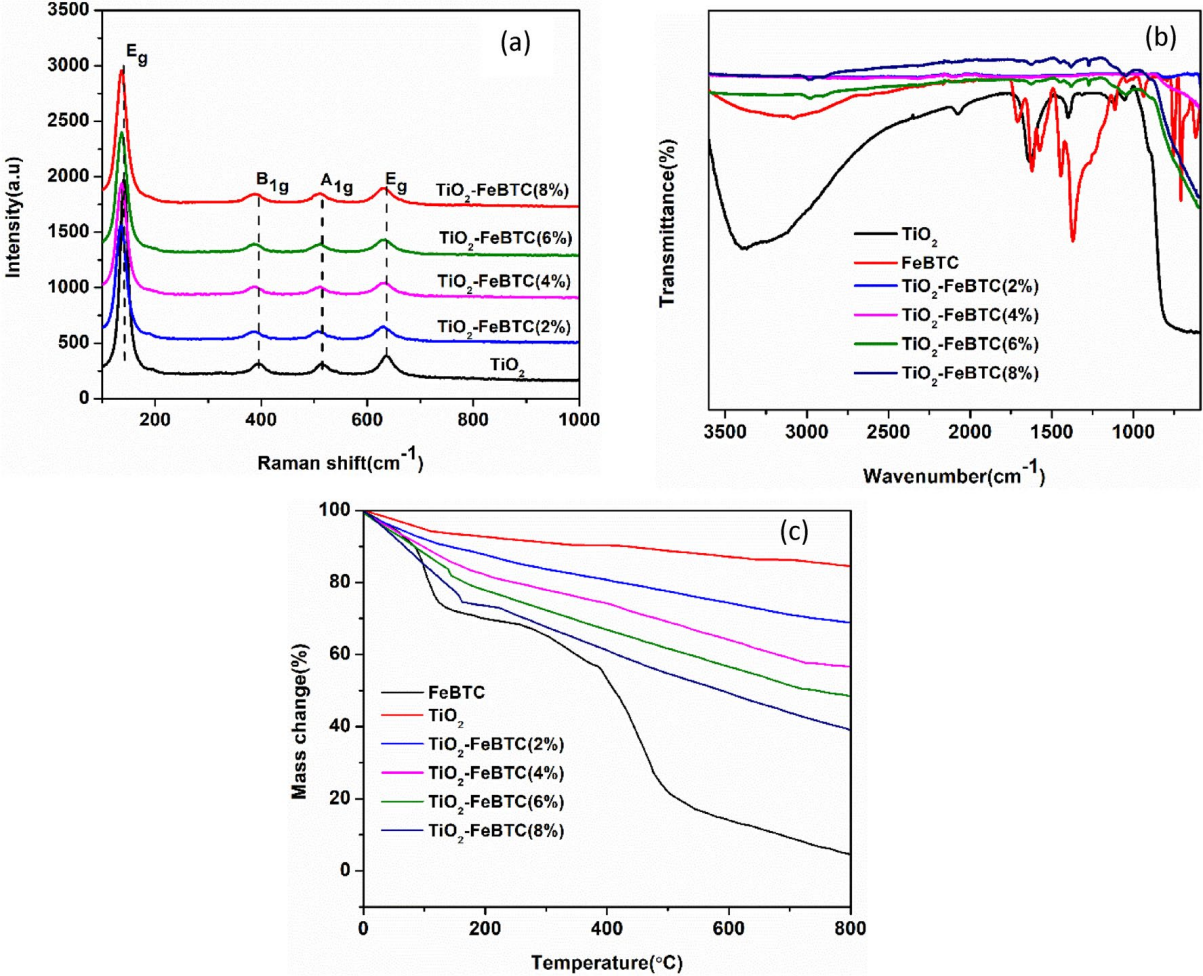
Shows the (**a**) FTIR, the (**b**) Raman spectra and (**c**) thermal gravimetric analysis of the as-synthesized materials.

The Raman spectra of pure TiO_2_ and TiO_2_–MOF nanocomposites (2, 4, 6 and 8%) is shown in Fig. 6a. The Raman spectrum of TiO_2_, demonstrated distinct Raman active modes of the E_g_ (142.7 and 635.6 cm^−1^), B_1g_ (396.5 cm^−1^) and A_1g_ (514.1 cm^−1^) which are characteristics of the anatase phase as also confirmed by XRD results. The Raman modes of anatase phase (E_g_, B_1g_ and A_1g_) are due to symmetric stretching vibration, symmetric bending vibration and the asymmetric bending vibration of O–Ti–O, respectively^34^. The E_g_ peak of anatase at 142.7 cm^−1^ decrease in intensity with an increase in Fe-BTC MOF loading, and there is blue-shift observed for all the E_g_, B_1g_ and A_1g of_ Raman modes in the TiO_2_–FeBTC (2, 4, 6 and 8%) nanocomposites. The increase in intensity is related to increase in crystallinity during the formation of the TiO_2_–FeBTC nanocomposites due to three-dimensional phonon confinement^35^. The shifting of Raman modes to lower wavenumber is related to an increase in crystal size of the TiO_2_–FeBTC (2, 4, 6 and 8%) nanocomposite materials^36^. In addition, the shifting in Raman peaks may be related to migration of electrons from the TiO_2_ nanoparticles which decrease the band strength within the O–Ti–O bond^12,37^. The change in Raman modes in all the nanocomposites spectra suggests that there is an interaction between the TiO_2_ and FeBTC MOF.

##### Analysis of nano-hybrids by surface functional groups composition

The FTIR spectra of the TiO_2_, Fe BTC MOF and TiO_2_–FeBTC MOF (2, 4, 6, and 8%), are shown in Fig. 6b. The TiO_2_ FTIR spectrum is characterized by bands observed between 3390 and 3560 cm^−1^ associated with the O–H stretching and bending vibrations of the water molecules adsorbed on the surface. The peak at around 1635 cm^−1^ is associated with the bending vibration of H–O–H group. The bending vibration of Ti–O–Ti in TiO_2_ is assigned to the broad peak^21,28^ at 540–800 cm^−1^. The spectrum of the FeBTC MOF is similar to the other reported by Delpiano et al. with the asymmetric stretching bands at 1629 and 1569 cm^−1^. The bands at 1456 and 1390 cm^−1^ were also observed which corresponds to the symmetric stretching of carboxylate groups in the BTC ligand^38^. It was observed that the TiO_2_–FeBTC MOF (2, 4, 6 and 8%) nanocomposite materials, demonstrated all characteristic bands which confirms the interaction of TiO_2_ and FeBTC MOF materials. It is worth noting that there is a decrease in intensity in TiO_2_–FeBTC (8%) nanocomposite due to increase in FeBTC concentration.

##### TGA analysis

To investigate the thermal stability of the as-synthesized materials, thermal gravimetric analysis (TGA) was performed as shown in Fig. 6c. The weight curve for Fe-BTC MOF shows several decomposition steps. At 100 °C, there is mass loss due to loss of moisture from water molecule. There is a mass loss due to destruction of the organic ligands was observed between 100 and 380 °C, while a total destruction of the Fe-BTC occurred from 400 to 550 °C, leaving only iron as the stable species. A minimal loss due to moisture below 100 °C was observed for TiO_2_, however, it remains stable throughout the temperature. The TiO_2_–FeBTC nanocomposites show relative stable curves compared to pure FeBTC MOF. However, as the Fe-BTC MOF loading is increased, the TiO_2_ becomes less thermally stable as evident by TiO_2_–FeBTC (2, 4, 6, and 8%) nanocomposites. This is induced by the less stable organic linker present in FeBTC MOF.

### Optical and electrochemical properties

#### UV–Vis spectroscopic analysis

**Figure 7:**
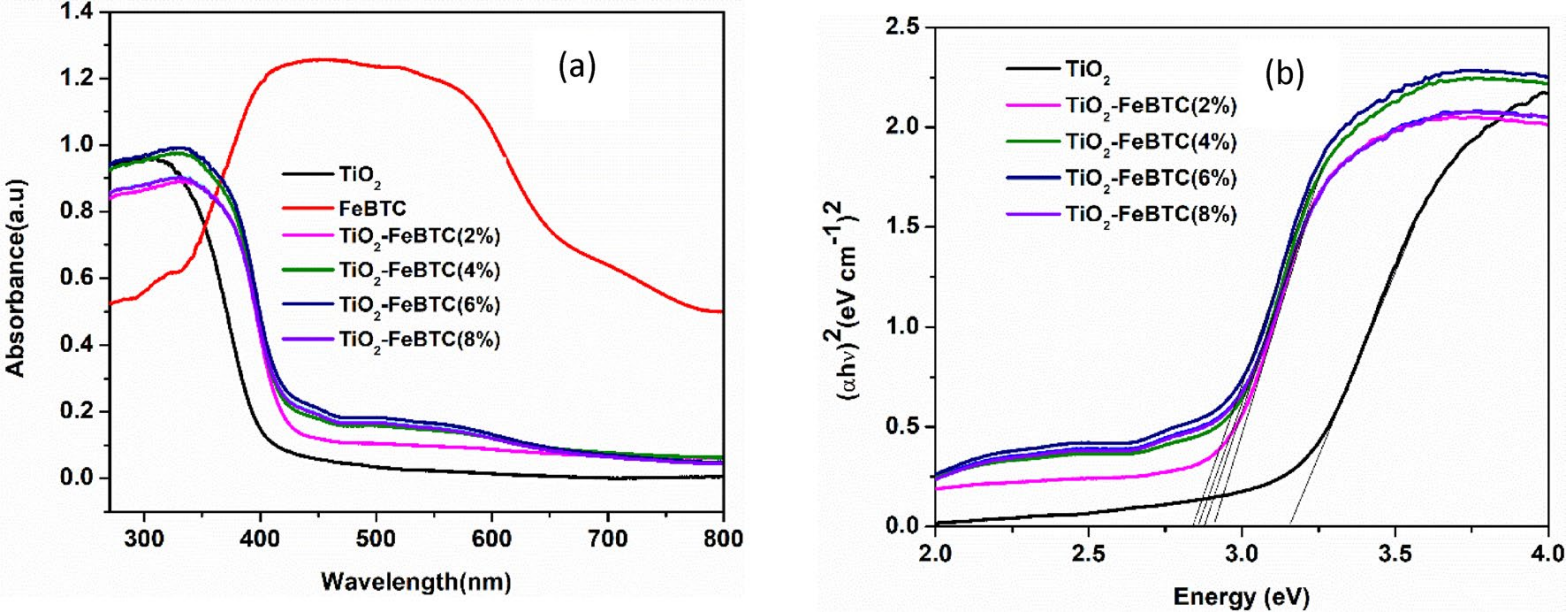
UV–Vis of TiO_2_ and TiO_2_–FeBTC MOF (2, 4, 6 and 8%) nano composites (**a**) and their corresponding Tauc plots (**b**).

Figure 7a, shows the UV–Vis diffuse reflectance spectra of pure TiO_2_, TiO_2_–Fe-BTC MOF (2, 4, 6, and 8%) nanocomposite materials. The pure TiO_2_ absorbs only in the UV region, with the absorption edge around 400 nm. The TiO_2_–FeBTC nanocomposites spectra are characterized by two absorption peaks with different intensity at 422 nm and around 550 nm. The intensity of these peaks increases with an increase in FeBTC MOF loading, with the TiO_2_–FeBTC (8%) showing the highest intensities. The appearance of the peak in the visible region suggest that there is an improvement in band gap energy which will allow TiO_2_ to absorb in this region. The Tauc plots in Fig. 7b confirmed that as the FeBTC MOF loading is increased, the bandgap energy narrows, which allows efficient charge transfer of electro-hole pairs and hence reduce recombination rate. This was expected as the FeBTC MOF has an intense broad peak in the visible region of 400–620 nm. This is desirable in the fabrication of DSSCs because it allows photoanode to absorb more light and improve the electron shuttling in the conduction band of TiO_2_, thereby improving the overall efficiency in the process.

#### Photoluminescence analysis

**Figure 8:**
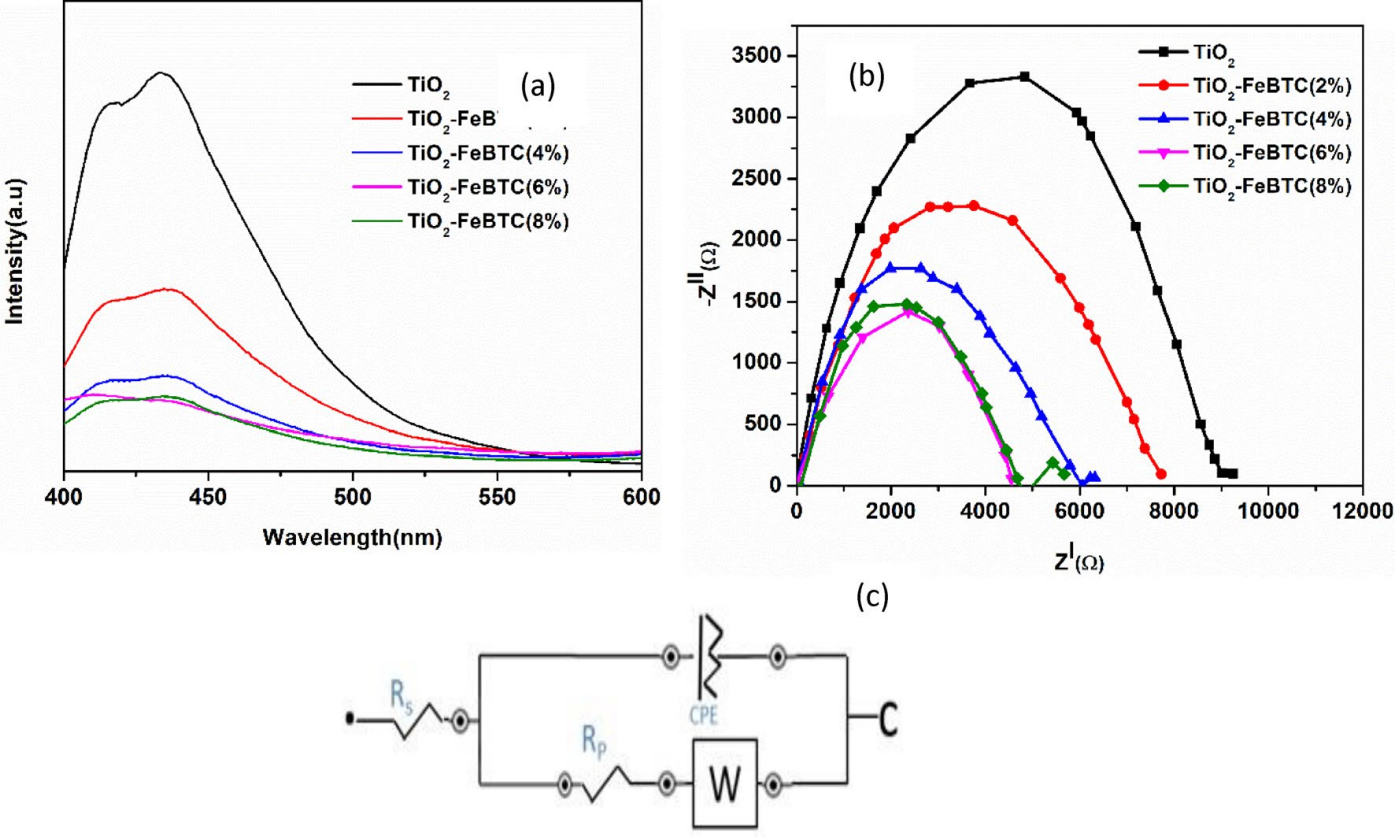
The EIS curves (**a**) and PL spectra (**b**) of the fabricated electrodes and (**c**) the Randel circuit used to fit EIS curves.

Photoluminescence (PL) was analysed to gain deeper insights into the photogenerated charge transport process in photocatalytic reaction by investigation of the efficiency of charge carrier trapping, transfer and migration and also understand the fate of electron–hole pairs in the as-synthesized nanocomposites. The recombination of the excited electrons and holes induce the PL emission. Under light irradiation, PL spectra was reported from excited electrons and holes, where the highest intensity correspond to higher recombination rate of electrons-hole pairs^39,40^. The excitation wavelength was at 325 nm for all the as-synthesized materials under this study. In Fig. 8a, pristine TiO_2_ has the higher intensity, which suggest that the material has the fastest recombination rate of holes and electrons relative to the FeBTC MOF and the nanocomposite materials. This indicate that the addition of FeBTC MOF reduced the recombination rate by bandgap engineering as suggested by UV–Vis studies. The addition of the MOF reduces the recombination rate of photogenerated charge carriers of electron–hole pairs. The 8% loading demonstrated a decrease in intensity which suggest an increase in recombination rate due to aggregation of FeBTC nanoparticles (Table 2). The increase in recombination rate may be related to the development of trapping sites which affects electron trapping in the shuttle process and therefore their development may greatly affect the performance of our materials in DSSCs.

#### EIS analyses for the TiO_2_, and TiO_2_–FeBTC MOF photoanodes

To further investigate the electron–hole pair separation behaviour of our materials, impedance studies were conducted on the prepared photoelectrodes. The photoanodes exhibited varied thickness of 17.26, 16.14, 14.11, 16.22 and 18.43 μm for TiO_2_, TiO_2_–FeBTC (2%), TiO_2_–FeBTC (4%), TiO_2_–FeBTC (6%) and TiO_2_–FeBTC (8%), respectively. EIS was conducted to investigate the charge transfer kinetics as shown with the Nyquist plots in Fig. 8b. The TiO_2_–FeBTC MOF nanocomposites were assessed, and the Nyquist plots are shown in Fig. 8b. The Randel circuit was used to fit the Nyquist curves and the equivalent circuit in Fig. 8c was used. Accordingly, the Rp values were 1572, 1351, 1174, 966 and 987 Ω for TiO_2_, TiO_2_–FeBTC (2, 4, 6 and 8%) nanocomposites. Based on the Rp values, there is a gradual decrease in charge resistance with an increase in Fe BTC MOF up to 6%. However, there is a sudden increase in charge transfer resistance when the loading is increased to 8%, which may be related to aggregation of TiO_2_–FeBTC nanoparticles, and thereby reducing the surface area and the pore size, which are the charge pathways and thus charge separation is compromised. This is supported by the SEM and BET results that showed that there is a decrease surface area due to increased aggregation. It was noted that the thickness of the photoanodes did not have correlation with the electron transportation. The decrease in charge transfer resistance with FeBTC MOF (6%) loading is due to the improved electron shuttling away from the hole in the TiO_2_ surface and thus retarding recombination rate of electron–hole pairs.

### Photoelectric performance of the TiO_2_–FeBTC MOF photoanodes

**Figure 9:**
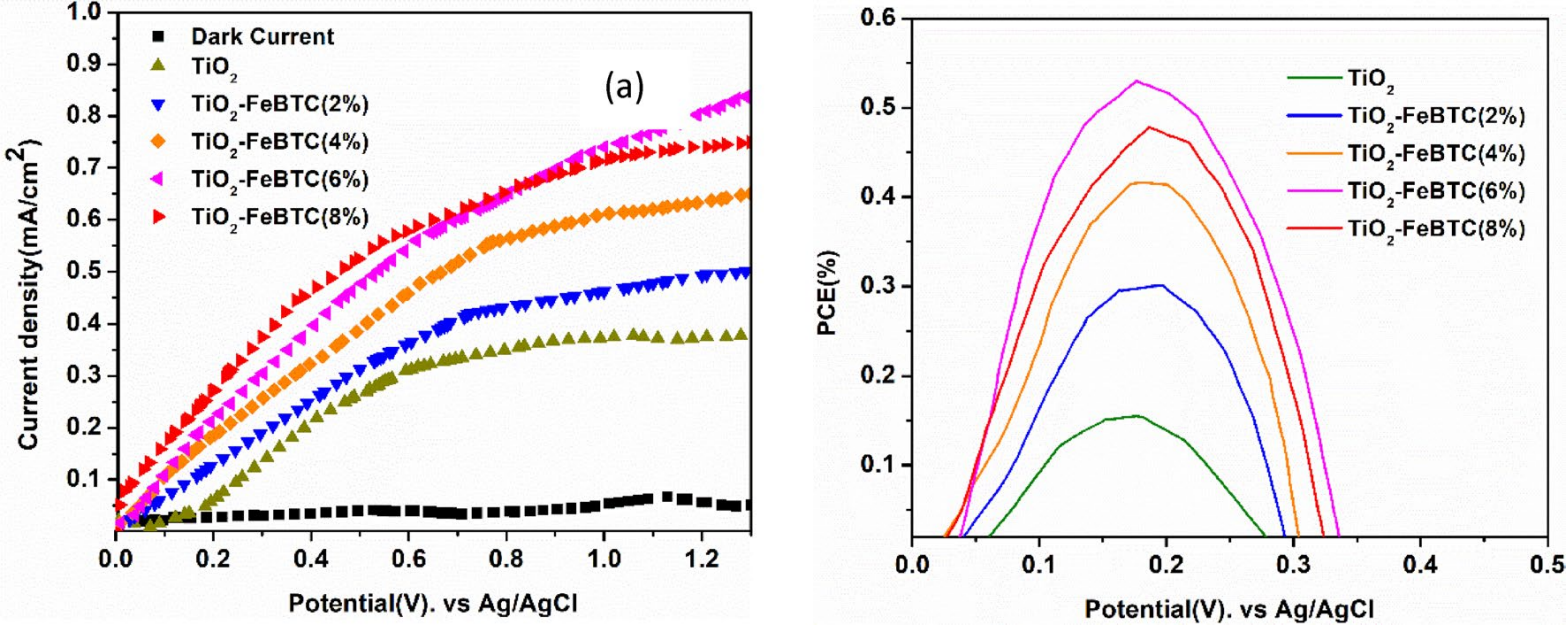
(**a**) I–V curves of TiO_2_ (**a**) and TiO_2_–FeBTC (2, 4, 6 and 8%)/thin film photoelectrodes and (**b**) the corresponding photocurrent conversion efficiency. The experiment was performed in a solution with 5 mM acetonitrile solution, 0.055 M LiI, 9 mM I_2_ and 0.85 M LiClO_4_ in the dark and under illumination using one-sun illumination (100 mW/cm^2^) and (**b**) shows the corresponding photo-conversion efficiency.

Photoelectric performance of the prepared thin film photoelectrodes are presented in Fig. 9a. Accordingly, under dark, the current was very low and therefore negligible. However, upon illumination, there was an increase in magnitude of current and again an increase in current with FeBTC MOF loading of the photoelectrode. The highest current was observed in the TiO_2_–FeBTC (6%) and was five times higher than that of pure TiO_2_. It is worth noting that beyond 8% loading of FeBTC MOF, there is a decrease in current generated. The charge resistance also starts to build up which inevitably increases the recombination rate. Power conversion efficiency as shown in Fig. 9b, was calculated from the I–V curves in Fig. 9a using the following equation:

$$PCE\%=\frac{{J}_{p}\left({E^\circ }_{rev}-{E}_{mea}-{E}_{cou}\right)}{{I}_{0}}\times 100\%$$

where, Jp is the photocurrent density (Am/cm^2^), E^0^_rev_ is the standard state-reversible potential (1.43 vs Ag/ AgCl) of the working electrode, E_cou_ is the open-circuit potential (vs Ag/AgCl) under the same working electrode experimental conditions, I_0_ is the power density of the incident light and E_mea_ is the electrode potential of the working electrode whose photocurrent was tested under irradiation.

As expected from the I–V curves, the loading of FeBTC MOF increased the photoefficiency of the photoelectrodes. The photoefficiency values of TiO_2_, TiO_2_–FeBTC (2, 4, 6 and 8%) nanocomposites were found to be 0.145, 0.298, 0.421, 0.562, and 0.497%, respectively. The TiO_2_–FeBTC (6%) loading, exhibited high photo conversion efficiency. In comparison with pure TiO_2_ photoanode, the photoefficiency of TiO_2_–FeBTC (6%) was over five times. These results are consistent with what we observed in the PL and EIS analysis. This suggest that TiO_2_–FeBTC (6%) can be a suitable candidate to use as a photoanode material in the development of DSSCs. To validate this work, the results were also compared to other reported TiO_2_ photoelectrodes as shown in Table 3. The TiO_2_–FeBTC (6%) photoefficiency was comparable with other developed electrodes and therefore can be suitable for use in DSSCs.

**Table 3:** The comparison of performance of other TiO_2_ modified photoelectrodes with the TiO_2_–FeBTC (6%) photoelectrode.

Photoanode	Method	R_P_ (Ω)	J (mA/cm^2^)	PCE (%)	Bandgap	Refs.
TiO_2_–Fe-BTC (6%)	Ultrasonic assisted method	966	0.87	0.538	2.85	Current
MIL(125)-NH_2_/TiO_2_ NRs	Hydrothermal	301	1.6	–	–	^41^
TiO_2_–WO_3_ nanotube	Electrodeposition	–	0.85	0.52	3.0	^42^
TiO_2_–Graphene	Hydrothermal	–	1.4	–	2.6	^43^
TiO_2_–ZnBTC (2%)	Ultrasonic-assisted method	540	1.01	0.67	2.8	^12^
TiO_2_/CdS	Electrodeposition	–	0.22	–	2.4	^44^
FeS_2_/TiO_2_	Wet chemistry	–	5.8	0.84	–	^45^
C@SiNWs/TiO_2_	Dip coating	23.9	5.56	1.17	–	^46^
Cd/TiO_2_	Hydrothermal	–	9.65	–	2.45	^47^

## Conclusions

This work reports the synthesis of TiO_2_–FeBTC nanocomposites through ultrasonic-assisted method and their subsequent fabrication into photoelectrode via spin coating technique. The surface, structural properties and optical properties were evaluated by XRD, FTIR, Raman, BET, TGA, FESEM, TEM, UV‒Vis DRS, and PL techniques. Enhanced surface area and high pore volumes were observed after incorporation of FeBTC MOF onto TiO_2_, which also improved charge transfer kinetics and reduced electron–hole recombination rates as confirmed by EIS and PL, respectively. The TiO_2_–FeBTC (6%) showed good charge separation of electron–hole pairs due to low charge resistance. The improved charge transport due to surface area has allowed the 6% loading to have excellence photoelectrical response as confirmed by I–V curves. The I–V results have showed that there is a direct proportional relationship between the generated photocurrent and FeBTC MOF loading. The optimal photocurrent density was observed in the TiO_2_–FeBTC (6%) at 0.87 mA/cm^2^. This resulted in high PCE value of 0.538% of TiO_2_–FeBTC (6%). This was attributed of relatively high surface area, which allowed more charge shuttling and thus better electrical response. Further increase in loading above 6% resulted in higher aggregation of TiO_2_ which gave rise to poor electron transport and thus higher recombination rate build-up. Therefore, it is necessary to optimise the loading of FeBTC MOF, as this can affect the performance of the photoanode in DSSCs. Based on the assessed performance of TiO_2_–FeBTC (6%), can form a viable candidate as a photoanode materials for DSSCs.

### Supplementary Information

Supplementary Figures.

## Data Availability

The datasets used and/or analysed during the current study available from the corresponding author on reasonable request.
