# Supplementary material for: Enhanced optical and electrochemical properties of FeBTC MOF modified TiO2 photoanode for DSSCs application
Source: Sci Rep. 2024 May 17;14:11292. doi: 10.1038/s41598-024-61701-3 (PMC11101415; doi:10.1038/s41598-024-61701-3)
Supplement: Supplementary file 1 — Supplementary Figures. [file 41598_2024_61701_MOESM1_ESM.docx]

**Figure** S1 O 1s spectra of TiO_2_ and TiO_2_-FeBTC(6%) showing the binding energy shift





**Figure** S2 Tauc plot for prestine FeBTC MOF
